# A phylogenetic analysis of the CDKL protein family unravels its evolutionary history and supports the *Drosophila* model of CDKL5 deficiency disorder

**DOI:** 10.3389/fcell.2025.1582684

**Published:** 2025-04-30

**Authors:** María del Carmen Martín-Carrascosa, Christian Palacios-Martínez, Máximo Ibo Galindo

**Affiliations:** ^1^ Instituto Interuniversitario de Investigación de Reconocimiento Molecular y Desarrollo Tecnológico (IDM), Universitat Politècnica de València, Universitat de València, Valencia, Spain; ^2^ Laboratory of Developmental Biology and Disease Mechanisms, Centro de Investigación Príncipe Felipe, Valencia, Spain; ^3^ UPV-CIPF Joint Unit Disease Mechanisms and Nanomedicine, Valencia, Spain

**Keywords:** cyclin-dependent-kinase like, CDKL5 deficiency disorder, epileptic encephalopathy, protein family, phylogeny, ciliogenesis, genetic redundancy

## Abstract

The human CDK-like (CDKL) family of serine‒threonine kinases has five members (CDKL1-5), with a conserved N-terminal kinase domain and variable C-termini. Among these, CDKL5 is of particular interest because of its involvement in CDKL5 deficiency disorder (CDD), a rare epileptic encephalopathy with several comorbidities for which there are no specific treatments. Current CDD vertebrate models are seizure resistant, which could be explained by the genetic background, including leaky expression of other CDKLs. Thus, phylogenetic analysis of the protein family would be valuable for understanding current models and developing new ones. Our phylogenetic studies revealed that ancestral CDKLs were present in all major eukaryotic clades and had ciliary/flagellar functions, which may have diversified throughout evolution. The original CDKL, which was likely similar to human CDKL5, gave rise to the remaining family members through successive duplications. In addition, particular clades have undergone further gene duplication and loss, a pattern that suggests some functional redundancy among them. A separate study focusing on the C-terminal tail of CDKL5 suggested that this domain is only functionally relevant in jawed vertebrates. We have developed a model of CDD in *Drosophila* based on downregulation of the single *Cdkl* gene by RNAi, which results in phenotypes similar to those of CDD patients, that are rescued by re-expression of fly *Cdkl* and human *CDKL5*. CDKL proteins contain a conserved kinase domain, originally involved in ciliary maintenance; therefore, invertebrate model organisms can be used to investigate CDKL functions that involve the aforementioned domain.

## 1 Introduction

The human CDKL (CDK-like) protein family has five members (CDKL1, CDKL2, CDKL3, CDKL4, and CDKL5) and belongs to the group of kinases named CMGCs after its main kinase superfamilies, CDKs, MAPKs, GSKs, and CLKs (Manning et al., 2002). The CDKL family is closest to the cyclin-dependent kinases (CDKs), which are serine‒threonine kinases, and structural studies indicate that they contain a conserved N-terminal kinase domain with a variable C-terminus (Canning et al., 2018; Malumbres et al., 2009). All five CDKLs contain the Thr-X-Tyr phosphorylation motif and possess putative cyclin-binding domains (Canning et al., 2018). Although their functions and interactions are still poorly understood, mutations in this family of proteins are responsible for a variety of conditions, such as epilepsy, carcinogenesis, tumour proliferation, defects in cognitive function and developmental disorders.

CDKL1 function has been related to ciliogenesis, cilia elongation, and Hedgehog signalling in primary cilia in *Caenorhabditis elegans* and zebrafish models (Hsu et al., 2011; Park et al., 2021). In addition, a role for this kinase in carcinogenesis and chemotherapy resistance has also been described (Li et al., 2019a; Li et al., 2019b; Sun et al., 2012).

CDKL2 is involved in normal behavioural control in mice in relation to the cognitive functions necessary to achieve contextual and spatial learning (Gomi et al., 2010). Furthermore, its deregulation has been shown to be associated with various types of tumours, such as gastric, breast and glioma, among others, as well as with increased mesenchymal‒epithelial transition (Li et al., 2014; Shao et al., 2020).

Although CDKL3 was initially associated with certain forms of mental retardation due to its involvement in the cell cycle and CNS development (Dubos, Pannetier, and Hanauer, 2008; Liu et al., 2010), it has since been linked to various types of tumours, such as squamous cell carcinoma, cholangiocarcinoma or osteosarcoma; however, in many cases, the pathways in which it participates are still not well defined (He et al., 2020; Midmer et al., 1999; Ye et al., 2019; Zhang et al., 2018).

Although little research has been conducted on this protein, according to high-throughput analyses, CDKL4 is found mostly in the testis, specifically in the spermatid development cycle, and less prevalently in the brain and other tissues. (The Human Protein Atlas, 2023, June 29; Fagerberg et al., 2014). A 2015 study linked the overexpression of this gene with an increased risk of both recurrence and death in colorectal cancer patients and reported that colon cancer cells died after CDKL4 was targeted in the presence of oxaliplatin (Lin et al., 2015). CDKL4 was identified as a candidate locus for behavioural responsiveness to stress in rats (Meckes et al., 2018).

The most studied family member is CDKL5, originally named serine/threonine kinase 9 (STK9) (Canning et al., 2018; Flax et al., 2024; Montini et al., 1998). This is because mutations in the *CDKL5* gene cause CDKL5 deficiency disorder (CDD). CDD (OMIM 300203, ORPHA: 505,652) is a rare developmental and epileptic encephalopathy that affects one in 42,000 births (Symonds et al., 2019) and is one of the most common types of genetic epilepsy in childhood. This disorder produces early-onset refractory epilepsy in infancy, which is mainly myoclonic and accompanied by other comorbidities, such as cortical visual impairment, hypotonia, global developmental delay, gastrointestinal problems, sleep disorders, impaired motor function and stereotypies (Leonard et al., 2022; Olson et al., 2019; Demarest et al., 2019). Although epilepsy is mainly myoclonic, absence seizures, partial seizures, tonic seizures and limb spasms have also been observed, which correlate with a wide range of abnormalities in electroencephalographic recordings (Daniels et al., 2024).

The genetic origin of CDD is due to *de novo* loss-of-function mutations in the *CDKL5* gene (Symonds et al., 2019). These mutations can be missense, nonsense or insertions/deletions. Since the gene is X-linked and disruption of both copies usually has fatal consequences for the individual, most patients are heterozygous females at a 4:1 ratio to hemizygous males; the few male patients who survive present genetic mosaicism (Jakimiec, Paprocka, and Smigiel, 2020; Van Bergen et al., 2022). Female patients are heterozygous, but at the functional level, they could also be mosaics due to random X chromosome inactivation. In addition, phenotypic differences have also been observed between sisters with the same mutation, suggesting that epigenetics and random inactivation of an X chromosome are crucial for the course of the disease (Jakimiec, Paprocka, and Smigiel, 2020; Olson et al., 2019).

The highest expression of CDKL5 coincides with the peri- and postnatal periods due to the rapid development of the nervous system, i.e., the cortex and hippocampus. Although it is distributed throughout the body, it is highly expressed in GABAergic and glutamatergic neurons of the hippocampus, striatum and cerebellum (Chen et al., 2010; Rusconi et al., 2008; Wang et al., 2012). The localization of the protein is diverse; it is located in the nucleus, cytoplasm, cilia, dendritic branches or other structures, depending on its function and stage of development. CDKL5, in contrast to other proteins in its family, has a long C-terminal domain with a putative regulatory function, as it can affect the localization and activity of the protein (Lin, Franco, and Rosner, 2005; Rusconi et al., 2008). For example, the cytoplasmic protein fraction plays a crucial role, as it is involved in the maintenance of dendritic spine structure and activity, whereas nuclear CDKL5 regulates RNA storage modification and splicing; therefore, it is involved in neuronal precursor proliferation, survival and neuronal migration. The importance of its various functions suggests that CDKL5 plays a key role in brain development (Jakimiec, Paprocka, and Smigiel, 2020; Olson et al., 2019; Ong et al., 2023; Valli et al., 2012; Zhu and Xiong, 2019). Some phosphorylation targets of CDKL5 have been identified, including the microtubule-associated proteins MAP1S, EB2 and ARHGEF2 and the calcium channel Cav2.3 (Baltussen et al., 2018; Sampedro-Castaneda et al., 2023).

Depending on where the mutation occurs, patients will experience more severe or milder symptoms (Diebold et al., 2014; Hector et al., 2017). On the one hand, the most severe cases involve missense mutations in the catalytic domain or frameshift mutations—due to point insertion or deletion—at the end of the C-terminal region. On the other hand, milder cases are those in which mutations occur in the ATP-binding region or nonsense mutations in the C-terminal region.

To understand the genotype‒phenotype correlation in such a complex pathology as CDD and to develop effective therapies, it is important to have informative models of the disease, including animal models. The most basic functions of CDKL5 proteins have been studied in the unicellular green alga *Chlamydomonas reinhardtii* (Tam, Ranum, and Lefebvre, 2013). The *LF5* gene, which codes for a protein that is most similar to human CDKL5, was identified because of its role in restricting flagellum length. This role seems to be conserved to some extent in mammals, since Cdkl5-deficient mouse hippocampal neurons have elongated primary cilia.

The first mouse knockout model was generated by inserting *loxP* sequences flanking exon 6, which was later renamed as exon 7, (Wang et al., 2012), such that deletion of this exon produced a premature stop codon and the absence of a functional protein. Hemizygous *Cdkl5* ^*-/y*^ males presented with behavioral, cognitive, and motor alterations and changes in protein phosphorylation patterns but no spontaneous seizure activity. Other groups have generated similar alleles that target the second and fourth exons (Amendola et al., 2014; Okuda et al., 2017). These mice have been tested in constitutive or conditional knockout experiments and consistently show defects in dendrite and axon morphology, synaptic connectivity, behaviour, and neuronal activity parameters; however, they do not display any spontaneous seizure activity (Amendola et al., 2014; Okuda et al., 2017; Tang et al., 2017; Schroeder et al., 2019). Spontaneous seizures have been described only in aged mice (Mulcahey et al., 2020; Terzic et al., 2021). Seizure activity is more common in heterozygous females than in homozygous females and hemizygous males, strongly suggesting that somatic mosaicism favours seizure susceptibility (Terzic et al., 2021). The only paper describing spontaneous seizures in 2- to 7-month-old males used a conditional deletion of *Cdkl5* in glutamatergic neurons (Wang et al., 2021). CDD models have also been generated in rats via CRISPR/Cas9 mutagenesis (Simoes de Oliveira et al., 2024). Hemizygous *Cdkl5* ^*-/y*^ males presented reduced hippocampal long-term potentiation but no spontaneous seizures.

Zebrafish have a single copy of the *cdkl5* gene, which is putatively orthologous to human *CDKL5*, which is expressed in the nervous system (Vitorino et al., 2018). A sense-shifted *cdkl5* mutant zebrafish line was generated through the Zebrafish Mutation Project via ENU chemical mutagenesis. Mutant fish exhibit abnormal craniofacial development, motor behaviour and neuronal branching but very mild seizure susceptibility (Serrano et al., 2022; Varela et al., 2022).

With respect to invertebrate models, the nematode worm *Caenorhabditis elegans* has a single *cdkl-1* gene, which is most similar to human CDKL1 and has the same function in regulating ciliary length (Park et al., 2021). Interestingly, introducing pathogenic *CDKL5* mutations found in CDD patients abolished this function as efficiently as a kinase-dead *cdkl-1* mutation (Canning et al., 2018).

One factor that could explain the apparent seizure resistance of vertebrate models could be genetic background, including leaky expression of other CDKL family members that could mask the phenotype. For the current model organisms, it would be necessary to consider how many *CDKL* genes they harbour and whether they could be functionally redundant. This information would also be relevant in the development of novel models that can complement the deficiencies of existing models.

Among invertebrate models, we have already explained the presence of a single *cdkl-1* gene in *C. elegans*. *Drosophila melanogaster* is a widely used model in the study of seizure disorders given the significant similarities between the fly and human nervous systems. *Drosophila* and humans can suffer different types of seizures, such as myoclonic, spasmic, tonic or tonic‒clonic seizures, and share several features regarding seizure phenotypes, such as having a seizure threshold, susceptibility to seizures being regulated by genetic mutations or response to antiepileptic drugs used in humans (Jacobs and Sehgal, 2020; Mituzaite et al., 2021; Parker et al., 2011). In *Drosophila,* there is a single putative homologous gene representative of the *CDKL1-5* human genes annotated as the candidate gene *CG7236*. Our preliminary work demonstrated that RNA interference against this gene causes seizures (data not shown), so it is a candidate model for CDD.

Hence, to understand the current models and support the development of new ones, it is necessary to understand the evolutionary history of this gene family and the gene complement in each species. Our phylogenetic analyses suggest significant functional equivalence among family members, which is corroborated by the ability of human CDKL5 to correct the phenotypes associated with deficiency in *Drosophila* Cdkl. We conclude that *Drosophila* is a relevant model for mutations affecting the kinase domain but not the C-terminal tail.

## 2 Materials and methods

### 2.1 Retrieval of CDKL protein sequences and multiple sequence alignment

Protein sequences were retrieved from the NCBI public database (https://www.ncbi.nlm.nih.gov/). We performed systematic BLASTP searches using the sequences of the five human CDKLs as queries. When we identified the homologous proteins in the species of interest, we performed the reverse procedure; each candidate was used as a query against human proteins and checked that the best match was a CDKL protein; and those that returned a different protein, usually a CDK, were discarded. The selected protein sequences were aligned using MUltiple Sequence Comparison by Log-Expectation (MUSCLE) (Edgar, 2004) through the Molecular Evolutionary Genetics Analysis (MEGA) software (www.megasoftware.net) (Tamura, Stecher, and Kumar 2021). The human CDK1 protein (NP_001307847) was used as an outgroup.

### 2.2 Kinase domain alignment and phylogenetic trees

The alignment was trimmed to the kinase domain with AliView (Alignment Viewer and Editor) software (Larsson, 2014), and the resulting alignment was realigned with MUSCLE. The gap penalty was set according to default parameters, as it gave an accurate alignment that was supervised and hand-corrected if necessary.

The Bayesian calculation phylogenetic tree was obtained with Metropolis-coupled Markov chain Monte Carlo (MCMC) methods via MrBayes 3.2.7 (Ronquist et al., 2012). In order to adjust the program to our data, some default parameters were modified. An analysis with four chains and two runs was chosen and was set to run for 3.5 × 10^7^ generations. A gamma-distributed rate variation across sites and a proportion of invariable sites (invgamma) were used to model the differences in evolutionary rates between sites. The amino acid model was adjusted to mixed and the JTT model was selected by the two independent runs (Jones et al., 1992). The model fitness was confirmed by both the minimum and maximum probabilities being 1.00 with a null standard deviation. As the two runs converged to a stationary distribution, the average standard deviation of the split frequencies was expected to approach zero, as the two tree samples were increasingly similar. The average standard distribution decreased from 0.05 in the 2.80 × 10^5^ generation to less than 0.01 in the 4.71 × 10^5^ generation, ending at 3.50 × 10^7^ generations at 4.238 × 10^−3^, a strong indication of convergence of the two analyses. This convergence of the analyses is also reflected in the “Effective Sample Size” parameter, which shows an appropriate ESS (>100), indicative of adequate mixing behaviour, and a Potential Scale Reduction Factor (PSRF) of 1.00, which shows a good sample from the posterior probability distribution. The analysis returned two trees, one specifying the probability of each clade in the tree and the other (phylogram) reflecting the branch lengths in expected substitutions per site.

To construct the maximum likelihood (ML) tree, all sites were considered, and an NJ tree (Saitou and Nei, 1987) was taken as a starting point for iterative searches using the LG model of amino acid substitutions (Le and Gascuel, 2008). A discrete Gamma (G) distribution was used to model the differences in evolutionary rates between sites (5 categories). This LG + G model was chosen because it was the best for all the datasets, according to the ML model comparison analyses available in MEGA 11. In the case of the neighbor‒joining tree, the JTT model was chosen. For the rates among sites, we opted for a Gamma distribution with a shape parameter of 5. Since it was not possible to choose all the sites, pairwise deletion was selected for the treatment of the gaps. The reliability of the phylogenetic reconstructions was estimated by bootstrap analysis with 1000 replicates for the ML tree and 500 replicates for the NJ tree.

### 2.3 CDKL5 proteins C-terminal tail alignment and phylogenetic trees

To study the conservation of the C-terminal tail of CDKL5, manual trimming of the original alignment was performed with AliView, leaving only the C-terminal region from the first amino acid after the kinase domain. In this alignment, we included all the proteins that were present in the CDKL5 branch of the previous tree, plus the two sequences from zoosporidic fungi. The resulting sequence set was realigned via MUSCLE through MEGA Software. The gap penalty was set according to default parameters, as it gave an accurate alignment which was supervised and hand-corrected if necessary.

The phylogenetic tree was obtained via Bayesian calculation with MCMC methods using MrBayes 3.2.7. The amino acid model was adjusted to mixed, and the JTT model was selected by the two independent runs. Its fitness was confirmed by both the minimum and maximum probabilities being 1.00 with a standard deviation equal to zero. A gamma-distributed rate variation across sites and a proportion of invariable sites (invgamma) were used to model the differences in evolutionary rates between sites. An analysis with four chains and two runs was chosen and was set to run for 2 × 10^7^ generations. The two independent runs converged after 5.73 × 10^6^ generations, with a standard deviation less than 0.01, and at the end of the 2 × 10^7^ generations, the analysis standard deviation was 5.409 × 10^−3^. The PSRF showed a good sample from the posterior probability distribution, with a value of 9.99 × 10^−1^. The analysis returned the clade credibility tree and the phylogram; some sequences appeared to form a polytomy, as the phylogenetic relationships between them could not be resolved.

### 2.4 Fly husbandry and genetics

The *Drosophila melanogaster* stocks and experimental crosses used in this work were cultured in standard cornmeal medium. All crosses were performed at 25°C unless otherwise stated. All the strains used in this work and their origins are listed in Supplementary Table S1.

### 2.5 Plasmid generation and transgenics

All the sequences of primers used in the molecular biology procedures are indicated in Supplementary Table S2. To construct the pUAS-attB-*Cdkl* plasmid, genomic DNA was extracted from Oregon flies using the Qiagen DNeasy Blood and Tissue Kit for DNA Isolation (Qiagen GmbH, Hilden, Germany). DNA was amplified via PCR using a GeneExplorer thermal cycler (BIOER, Hangzhou, China) with primers with adapters for the pUAS-attB vector (stock n° 1419, *Drosophila* Genomics Resource Center, Indiana University). The vector and the PCR product were double digested with the restriction enzymes *NotI* and *XbaI* (Thermo Fisher Scientific, Waltham, Massachusetts, USA), ligated, and transformed into DH5α competent cells (Thermo Fisher Scientific, Waltham, Massachusetts, USA). The construct was validated by Sanger Sequencing by means of the 3730xl DNA Analyzer (Applied Biosystems, Waltham, Massachusetts, USA).

Human *CDKL5* cDNA (courtesy of Navjot Pabla, Ohio State University) was extracted from the pCMV6 vector by PCR. The pUAS-attB plasmid was cut by digestion with the *BglII* and *XbaI* enzymes (Thermo Fisher Scientific, Waltham, Massachusetts, USA), and the cDNA of *CDKL5* was cloned via the ClonExpress UItra One Step Cloning Kit (Nanjing Vazyme Biotech Co., Nanjing, China). Clones were validated as explained above.

For the generation of the transgenic lines, the purified plasmids were sent to the *Drosophila* Transgenesis Service of the Centro de Biología Molecular Severo Ochoa (CSIC-UAM, Madrid, Spain). The vectors carrying the attB insertion sequence of the Phi-C31 phage were injected into embryos of strains carrying independent attP sequences on chromosome II (2R-51D) and chromosome III (3R-86F). Transgenic flies were selected for the presence of the mini-white eye colour reporter. To ensure that they contained the correct gene, the genomic DNA was extracted, amplified by PCR and sequenced as described above. The expression of the transgenic constructs was validated by crossing them with the *elav-Gal4* driver and performing RT‒PCR with RNA extracted from the heads of the progeny (see below). For this test, a forward primer targeting the integration site and a reverse primer targeting the integrated construct were used.

### 2.6 RT‒PCR and qPCR

For each biological condition, three replicates with 40 adult male fly heads were assayed. Flies were beheaded in groups of 40 and rotated overnight at 4°C in a 1.5 ml microcentrifuge tube (ASTIK’s, Labbox Labware, Barcelona, Spain) with the stabilization solution RNa*later* (Invitrogen, Massachusetts, USA). The next day, RNA extraction was performed with an RNeasy Mini Kit (QIAGEN, Hilden, Germany). First, the 40 heads were transferred into Lysing Matrix A tubes (MP Biomedicals, California, USA), and TRIzol (Thermo Fisher Scientific; Waltham, Massachusetts) was added; they were homogenized in a Mini-Beadbeater-24 (BioSpec Products, Bartlesvile, Oklahoma) for 30 s at 3800 rpm, and the protocol from the RNeasy Mini Kit was followed. Rests of DNA were cleaned with DNaseI Amplification Grade (Thermo Fisher Scientific, Waltham, Massachusetts, USA). cDNAs were subsequently generated with the qScript cDNA SuperMix Kit (Quantabio; Beverly, Massachusetts).

For the qPCR experiments, we used FastStart Essential DNA Green Master Mix (Roche; Mannheim, Germany) and the LightCycler 480 (Roche; Bassel, Switzerland). The expression analysis was performed via the 2^−ΔΔCT^ algorithm. The samples were normalized by comparing their expression to that of three constitutive genes: *RpL32*, *FoxK/Mn*f, and *eEF1α1*.

### 2.7 Seizure phenotypes

In all the tests for seizure phenotypes, groups of 12 male flies were introduced into empty plastic vials 20 min before the beginning of the assay; each group constituted a biological replicate. For each experiment, six biological replicates were performed, each of which was assayed three times. Loss of posture and wing buzzing were identified as seizures, and phenotypes were scored as the number of episodes, bearing in mind that one individual can seize more than once.

For spontaneous seizures, vials were placed sideways on a white background under zenithal light, three videos of three minutes were taken per biological replicate, and the videos were scored for spontaneous seizures. For the mechanically induced seizure test, the vials were vortexed at maximum speed (40 Hz) for 10 s, the process of recovery was recorded on video, and seizures were assessed over the following 10 s. For the heat-induced seizure test, the vials were placed for 2 min in a CORIO CD water bath with transparent plastic walls (JULABO GmbH, Seelbach, Germany), with the temperature set at 40°C ± 0.5°C, ensuring that the hot water completely covered the walls of the tube. The behaviour of the flies in the submerged vials was continuously videotaped during the 2-min immersion.

### 2.8 Lifespan assays

Twenty male individuals of each experimental genotype were placed in a vial of standard cornmeal medium. Every 3–4 days, the number of deaths was scored, and the surviving individuals were transferred into tubes with fresh food. Two biological replicates were performed for each genotype.

### 2.9 Negative geotaxis

Twelve male flies were transferred into empty vials and left to settle for 20 min. Previously, these empty vials were marked with a line 80 mm from the base. The vials were tapped against a cork pad onto the laboratory bench to knock down all the individuals to the bottom of the tube, and they were video recorded as the flies tried to climb back to the top of the vial. The videos were examined to assess how many flies were able to climb pass the 80 mm mark in 10 s. There were three biological replicas and three experimental replicas, allowing half an hour between experiments. The performance index was defined as the proportion of individuals (%) able to cross the mark.

### 2.10 Flight test

The experimental design was adapted from previously published studies (Babcock and Ganetzky, 2014). We constructed a methacrylate flight tester tube 100 cm in height and 12 cm in diameter with an inner sheet of PVC plastic coated with adhesive glue for rodent traps (Cofan, Ciudad Real, Spain) to determine how much time flies need to produce enough thrust with their wings to stabilize and get stuck to the wall. A “drop tube” was incorporated to guarantee that the flies would enter the flight test device at the same rate, thereby reducing the variability associated with manipulation.

A total of 35 flies from three biological replicates were used. First, the flies were left to settle for 20 min in an empty vial. The flasks were subsequently dropped into the flight tester via the drop tube. Afterwards, the plastic sheet was removed, unrolled, placed on a flat surface and photographed. The images were analysed using theImageJ2 software (Rueden et al., 2017). If the flies dropped to the bottom of the flight tester, the scored distance was 100 cm. The flies attached to the initial protective tape could not be by ImageJ2 software, and they were assigned a distance of 1 cm.

### 2.11 Locomotion

The method was adapted from previously published studies (Stone et al., 2014). Individual flies were introduced into a circular arena enclosed by an inverted 90 mm Petri dish (Thermo Fisher Scientific Cat. No. 101/IRR) and left to settle for 20 min. The Petri dish was placed on top of a dim white led transilluminator. Using a webcam, a 10 min video was recorded per fly using the HandyAvi software (https://www.azcendant.com). Individual photograms were saved at a rate of 0.2 s/frame and uploaded to ImageJ software. The spatial coordinates of each fly in the arena were calculated and introduced into an Excel spreadsheet to calculate the average speed and total distance. In addition, an inner area, 50 mm in diameter and concentric with the larger arena, was defined to determine the proportion of time spent in the central and peripheral regions of the Petri dish. For each genotype, 15 male individuals were used.

### 2.12 Data analysis

The data analysis was performed by combining the software GraphPad Prism 10.2.3 (GraphPad Software, Inc., California) and Excel 2021 (Microsoft Corporation, Albuquerque, New Mexico). The normal distribution of the dataset was analysed applying Shapiro-Wilk test as the sample size was less than 50. When the sample was composed of a maximum of two independent groups adjusted to a Gaussian distribution, an unpaired t-test was used for pairwise comparisons. However, if the results did not follow a normal distribution, the Mann‒Whitney test was used. In the cases with more than two independent groups, one-way ANOVA followed by Šídák’s multiple comparisons test or Dunnett’s multiple comparisons test were applied, depending on whether we compared a set of means or every mean with a control mean, respectively. For the lifespan analysis, a log-rank (Mantel‒Cox) test was chosen. In every analysis, the statistical significance was measured with a two-tailed P-value parameter with a 95% confidence interval (α = 0.05).

## 3 Results

### 3.1 Phylogeny of the CDKL proteins based on the kinase domain

When we look at the complement of CDKL proteins in current and candidate CDD animal models, the landscape is very heterogeneous, including species with one (*D. melanogaster*, *C. elegans*), two (*D. rerio*) or five (mammals) family members. In order to answer questions such as whether the lack of phenotype in murine models is due to functional redundancy or if invertebrate models could be a good alternative; it would be helpful to obtain a more comprehensive picture of the dynamics of this protein family during evolution.

For the construction of the phylogeny, we selected a set of species covering the main branches of the eukaryotes, but since our overarching interest is on the existing and candidate disease models, the emphasis was placed on metazoan species. Among the non-metazoans, we included the groups of Viridiplantae (*Chlamydomonas reinhardtii, Arabidopsis thaliana*), Ascomycota (*Saccharomyces cerevisiae*), *C*hoanozoa (*Monosiga brevicollis, Capsaspora owczarzaki*) and Ciliophora (*Tetrahymena thermophila*). Among metazoans, we started with the most basal animals, including sequences of placozoan species (*Trichoplax adhaerens*) and a cnidarian (*Nemastostella vectensis*). Among the protostomes, we included sequences of the two main branches, Lophotrochozoa and Ecdysozoa. From the Lophotrochozoa, we selected the mollusc *Lottia gigantea,* and from the Ecdysozoa different arthropods (the crustacean *Daphnia pulex* and the insects *Drosophila melanogaster* and *Apis mellifera*), and the nematode worm *Caenorhabditis elegans*. Among the deuterostomes, we included a hemichordate (*Saccoglossus kowalevskii*) and several chordates: a cephalochordate (*Branchiostoma floridae*), a tunicate (*Ciona intestinalis*) and six vertebrate species (*Danio rerio*, *Xenopus tropicalis*, *Caretta caretta*, *Gallus gallus*, *Mus musculus* and *Homo sapiens*).

For the identification of CDKL protein sequences, we used BLAST searches, and those with high similarity and quality were verified via reverse BLAST. In this search, we failed to identify CDKL sequences in several non-metazoan species, such as *A. thaliana*, *S. cerevisiae* and *C. owczarzaki*. In *S. kowalevskii,* we identified three CDKL sequences, putatively similar to CDKL1, CDKL2 and CDKL5, but the last one had an insertion of 30 amino acids within the kinase domain. This sequence is a predicted annotation without experimental support, and the corresponding locus is located within a low-quality genomic contig with several large gaps. Therefore, we decided not to use the sequences from this species, since the insertion in CDKL5 could distort the phylogeny. There are no alternative hemichordates with a complete genome sequence and annotation.

Our preliminary alignments revealed, as expected from previous analyses, that CDKL proteins have a highly conserved N-terminal kinase domain and C-terminal regions of variable length and very poor conservation (Canning et al., 2018; Li et al., 2024). Therefore, to construct a more robust phylogeny, we decided to align only the kinase domains. To ensure that the topology was consistent, three phylogenetic trees were constructed via different methods: maximum likelihood (ML), neighbor joining (NJ) and Bayesian methods. All three methods produced trees with very similar topologies (data not shown).

This phylogenetic analysis suggested a pattern of gain and loss of *CDKL* genes in different groups, so to obtain a more comprehensive picture, we decided to include CDKL sequences from additional species. The most striking finding was that although CDKLs seem to be present in all major eukaryotic clades, such as Ciliophora, Plants and Opisthokonta (including fungi and animals), CDKLs seem to have been lost in green plants, yeast and Filasterea. Filasterea are a group of protists closely related to animals (Shalchian-Tabrizi et al., 2008). What all these groups have in common is the loss of cilia and flagella. This finding suggests that ancestral CDKL proteins were intimately linked to ciliary/flagellar functions. These organelles were present in the last eukaryotic common ancestor (LECA) and have been lost independently in some fungi, Amoebozoa (*Dictyostelium*), and angiosperms (Carvalho-Santos et al., 2011). To test this hypothesis, we performed a BLAST search with the CDKL protein sequences against the kingdom Fungi. Most fungi have lost their flagellum, with the exception of some unicellular fungi that are zoosporidic; that is, they reproduce through flagellated spores (Galindo et al., 2021). Our BLAST search revealed CDKL proteins only in species belonging to two phyla of these zoosporidic fungi, *Blastocladiomycota* (*Paraphysoderma sedebokerense*, *Allomyces arbusculus*) and *Chytridiomycota* (*Dinochytrium kinnereticum*, *Chytridiales* sp. *EL0842*, *Phlyctochytrium bullatum*, *Boothiomyces* sp. *JEL0838*, *Thoreauomyces humboldtii*, *Rhizophlyctis rosea*). In addition, BLAST against the genus *Dictyostelium* failed to identify any CDKL proteins, only CDKs. These results support the ancestral ciliary/flagellar role of CDKL proteins, some of which still maintain it to some extent, although their functions may have diversified during evolution. Therefore, we included species representing the two zoosporidic fungal phyla, *A. arbusculus* and *D. kinnereticum*.

Another inconsistency was that *D. melanogaster* has a single CDKL protein, similar to other Ecdysozoa, with the exception of *A. mellifera*, which has two CDKL proteins. To clarify the situation in insects, we searched for CDKL sequences in several orders and identified a second CDKL in evolutionarily distant insects such as ephemera, termites, cockroaches, beetles and moths, which means that ancestral insects experienced duplication of the original CDKL and that this second protein was lost in Diptera. This second CDKL is also absent in the rest of Diptera. Therefore, we included the CDKL sequences of a third insect species, the beetle *Tribolium castaneum*.

Although most animal species consistently have three CDKLs, similar to CDKL1, CDKL2 and CDKL5, among the vertebrates, there is great variability, with five CDKLs in mammals, two or three in fish (zebrafish and eel, respectively), four in reptiles and amphibians, and three in chickens; thus, we explored further species in these and related classes. Lampreys (Hyperoartia), the most ancient fish, maintain the basic set of three CDKLs, but one of them has experienced duplications. There are two species with good-quality genomic data: *Petromyzon marinus* has undergone two duplications (five copies), and *Lethenteron reissnieri* has undergone a third duplication, which is a tandem duplication within chromosome 60 (six copies). Assuming that this duplication may be unique to this species, we included the sequences from *P. marinus* in our set.

Among Chondrichthyes (cartilaginous fish), there are species such as *Heptranchias perlo* (sevengill shark) that already have six CDKLs putatively similar to each of the five mammalian proteins, with one class duplicated. Therefore, the ancestor of cartilaginous fish, bony fish and tetrapods must have had the full set of CDKLs. Among bony fish, zebrafish and eel had two and three CDKLs, respectively, but further BLAST searches revealed that species as divergent as trout, sturgeon and coelacanth had a fourth CDKL. Therefore, we included in our set the sequences of the shark *H. perlo* as representative of cartilaginous fish and of the trout *Salmo trutta* as representative of the majority of bony fish.

*X. tropicalis had* four CDKLs, but searches against amphibian species revealed that a fifth CDKL was present in several species of the limbless Gymnophiona order and in a single frog, *Ascaphus truei*, from the suborder Archaeobatrachia, a group of primitive frogs sister to all the other frogs; although one of the *A. truei* sequences was partial, some N-terminal aminoacids were mised. BLAST searches failed to detect this fifth CDKL in other Anura (frogs and toads). We also searched against Urodela, tailed amphibians, but this search was complicated since these animals have very large genomes, with scarce and poorly annotated sequences (Pyron et al., 2024; Kosch et al., 2025). As previously mentioned, one of the *A. truei* sequences was incomplete, but we could not find any other Archaeobatrachia with better genomic sequences; therefore, we decided to include this species in our analysis, together with the CDKL sequences of the Gymnophiona *Rhinatrema bivittatum.*

*G. gallus* has only three CDKLs, and searches against all avian species revealed that the same was true for most of them, except for Palaeognathae (ostriches, kiwis and tinamous), which has a fourth CDKL. From these, we chose the CDKL sequences from North Island brown kiwi (*Apteryx mantelli*) for inclusion in our set. For the last two classes of tetrapods, reptiles and mammals, we found that they were well represented by the original species, since in-depth searches did not reveal any further gain or loss of CDKL classes. The final set of species providing the sequences used in the phylogeny is indicated in Table 1.

**TABLE 1:** Metazoan and nonmetazoan species for phylogenetic analyses.

	Species	Phylum; class
Nonmet.	*Tetrahymena thermophila* (M)	Ciliophora; Oligohymenophorea
	*Chlamydomonas reinhardtii* (M)	Viridiplantae; Chlorophyta
	*Dinochytrium kinnereticum*	Chytridiomycota; Chytridiomycetes
	*Allomyces arbusculus*	Chytridiomycota; Blastocladiomycetes
	*Monosiga brevicollis*	Choanozoa; Choanoflagellatea
Metazoan	*Trichoplax adhaerens*	Placozoa; Trichoplacoidea
	*Nemastostella vectensis*	Cnidaria; Anthozoa
	*Carnorhabditis elegans* (M)	Nematoda; Secernentea
	*Lottia gigantea*	Mollusca; Gastropoda
	*Daphnia pulex*	Arthopoda; Branchiopoda
	*Drosophila melanogaster* (M)	Arthropoda; Insecta
	*Apis mellifera*	
	*Tribolium castaneum* (M)	
	*Ciona intestinalis*	Chordata; Ascidiacea
	*Branchiostoma floridae*	Chordata; Cephalochordata
	*Petromyzon marinus*	Chordata; Hyperoartia
	*Heptranchias perlo*	Chordata; Chondrichthyes
	*Anguilla anguilla*	Chordata; Actinopterigii
	*Danio rerio* (M)	
	*Salmo trutta*	
	*Rhinatrema bivittatum*	Chordata; Amphibia
	*Ascaphus truei*	
	*Xenopus tropicalis*	
	*Caretta caretta*	Chordata; Reptilia
	*Apteryx mantelli*	Chordata; Aves
	*Gallus gallus* (M)	
	*Mus musculus* (M)	Chordata; Mammalia
	*Homo sapiens*	

(M) indicates those species that have been used to study CDKL function or that are commonly used as biological or biomedical models.

**FIGURE 1:**
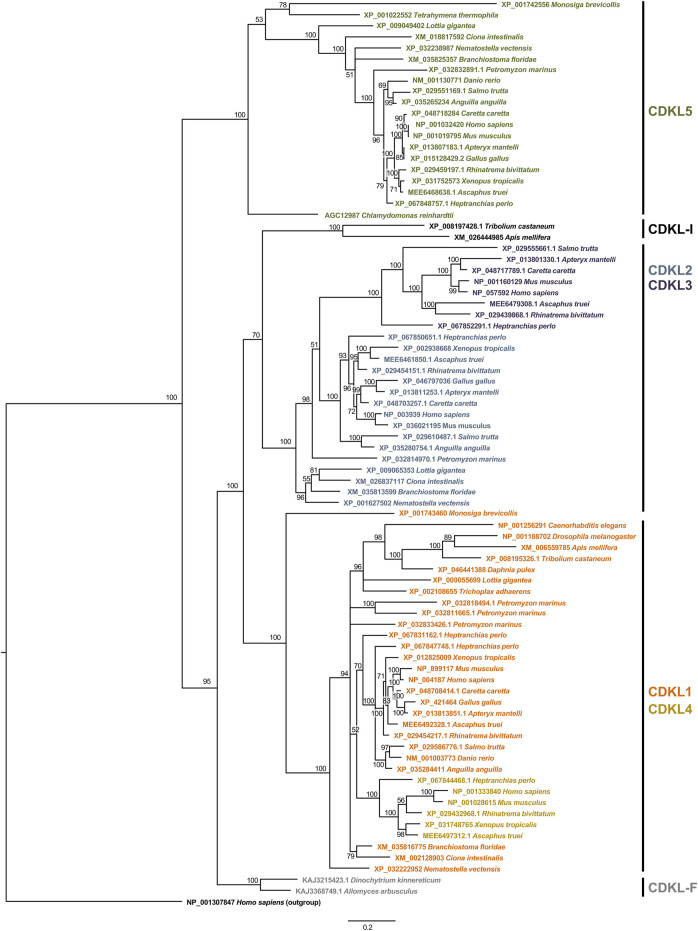
Phylogeny of the kinase domain of CDKL proteins. The aligned sequences of the kinase domain of CDKL proteins were used to construct a phylogenetic tree by the Bayesian method. Branch lengths are proportional to the amount of genetic change measured as substitutions per site; the scale bar is shown below the tree. For each branch, the bootstrap statistical support is indicated as a percentage. For each sequence, the accession number and species are indicated. On the right, we indicate the protein families defined on the basis of the phylogeny.

After alignment and trimming of the new set of sequences, we recalculated the phylogenetic trees via the same three methods: Bayesian, NJ and ML. From these, we chose the Bayesian tree as the reference, since it has more robust probability values between nodes, among other statistical parameters (Figure 1). In the ML and NJ trees, the topology of the main clades remained unchanged, and there were small differences in minor clades, but they did not significantly alter the phylogeny (Supplementary Figures S1, 2). However, these two trees present a lower statistical reliability than the Bayesian phylogeny does, since several nodes, especially those that do not group chordates, present low bootstrap values.

In this reference tree, the first split is between the branch containing CDKL5 and the rest of the CDKLs. The CDKL5 branch contains sequences from species belonging to very diverse eukaryotic groups, such as Opisthokonta, Ciliophora and Chlorophyta, so a protein most similar to CDKL5 must have been present in ancestral eukaryotes and would be the founder member of the family. However, it was lost in some groups throughout the Eukarya domain, such as Placozoa and Ecdysozoa.

In the other branch, two other groups split almost simultaneously to form the CDKL1/CDKL4 and CDKL2/CDKL3 branches. The odd-one-out among these sequences would be a CDKL present in insects, with the exception of Diptera, which does not clearly group with either of these two more recent branches and may represent a rapidly evolving protein. For these proteins, we propose the name CDKL-I. The CDKL1/CDKL4 branch contains proteins from metazoans and choanoflagellates, and it includes the protein encoded by *Drosophila CG7236* and CDKL4 in fish and tetrapods. In the other branch, CDKL2 is present only in metazoans, although it has been lost in Ecdysozoa, and CDKL3 is present in fish and tetrapods, although it is absent in some species of fish, amphibians and birds.

One indetermination in this tree is the origin of the CDKLs of zoosporidic fungi. Although the most ancestral protein seems to be related to CDKL5, the fungal sequences group with the remaining CDKLs in a separate branch. Since they do not group with any of the major branches, we named these sequences CDKL-F.

### 3.2 Evolutionary history of the CDKL protein family

**FIGURE 2:**
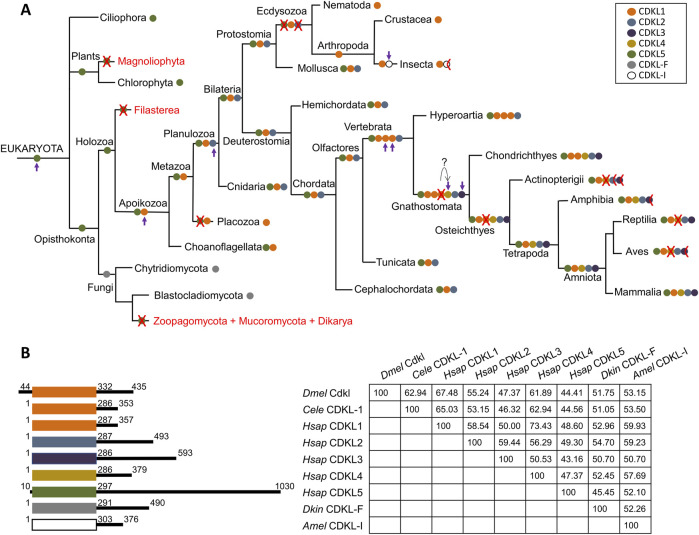
Proposed evolutionary history of the CDKL protein family. **(A)** CDKL proteins are color-coded according to their class. A red cross indicates the loss of a CDKL class in all species of a branch, and a half cross indicates loss in some species only (see text for more details). Complete lack of CDKL proteins is indicated by the red type. The appearance of new families is marked by a purple arrow. **(B)** Cartoon of CDKL proteins representative of the main classes color-coded as above, with kinase domains aligned; coordinates indicate the first and last amino acids of the kinase domain, and the total size of the protein; and amino acid identity matrix for the kinase domain. *Dmel*: *D. melanogaster*, *Cele*: *C. elegans*, *Hsap*: *H. sapiens*, *Dkin*; *D*. *kinnereticum*, *Amel*: *A. mellifera*.

In light of the previous analyses, an evolutionary history of the CDKL protein family can be inferred (Figure 2A). LECA has a protein with a kinase domain similar to that of CDKL5 with ciliary function, which is lost in angiosperms, nonflagellated fungi and Filasterea. The phylogeny did allow a clear assignment of the sequences of zoosporidic fungi, CDKL-F, to one of the major classes; the most parsimonious explanation would be that they are derived from the ancestral CDKL5-related sequence, but alternatively, it is possible that this new branch formed in the ancestor of Opisthokonta, after which CDKL5 was lost in these fungi.

In the Apoikozoa, the CDKL1 class appeared as a duplication of CDKL5, and CDKL5 was lost in the Placozoa phyla. Later, CDKL2 appeared as a duplication of CDKL1 in Planulozoa, and this basic set of CDKL1/CDKL2/CDKL5 genes was retained for most animal evolution. Strikingly, Ecdysozoa lost both CDKL2 and CDKL5, leaving only CDKL1; however, insects experienced further duplication, and the new CDKL-I protein accumulated rapidly, suggesting an adaptation to a new function. This protein was lost in Diptera.

Both lampreys and cartilaginous fish had more than one CDKL1 gene, so this amplification may have started in earlier vertebrate ancestors. While lampreys had three CDKL1s (assuming that the tandem duplication in *L. reissnieri* was exclusive to this species), cartilaginous fish had two CDKL1s and the novel CDKL4. It is tempting to speculate that this CDKL4 originated from one of the triplicated CDKL1s, but they may have been due to independent gene duplication and differentiation events. At the same time, CDKL3 must have originated from CDKL2, since it was present in both branches originating from Gnathostomata. Therefore, cartilaginous fish had the full complement of five CDKLs, plus one duplicated CDKL1, and ray finned fish (Actinopterygii) and tetrapods lost the duplicated CDKL1, so these groups have a maximum of five CDKLs.

Among the Actinopterygii, most have four CDKLs due to the loss of CDKL4, whereas CDKL3 would have been lost in Anguilliformes, and both CDKL2 and CDKL3 would have been lost in zebrafish. Zebrafish belong to the order Cypriniformes, and searches with human CDKLs revealed the absence of CDKL3 proteins but the presence of CDKL2 in most families of Cypriniformes; thus, the loss of CDKL2 would be exclusive to this species/genus. The order Anguilliformes is very distant from Cypriniformes, so the loss of CDKL3 would have occurred independently in both orders.

In Amphibia, all five proteins were present, but CDKL3 was absent at least in Neobatrachia; CDKL4 would have been lost both in reptiles and birds, and CDKL3 was lost in Neognathae but not in Palaeognathae birds. Therefore, among tetrapods, only gymnophiona amphibians and mammals would have one representative of all five CDKL classes.

According to the previous analyses, *D. melanogaster CG7236* belongs to the CDKL1 class, together with *C. elegans* CDKL-1 and human CDKL1 among others (Figure 2B). Consistent with this, the identity matrix shows greater conservation with CDKL1s and the related CDKL4s. In contrast, the C-terminal portions of the proteins represented are highly heterogeneous in size and sequence.

### 3.3 Phylogeny of the C-terminal regions of CDKL5 proteins

**FIGURE 3:**
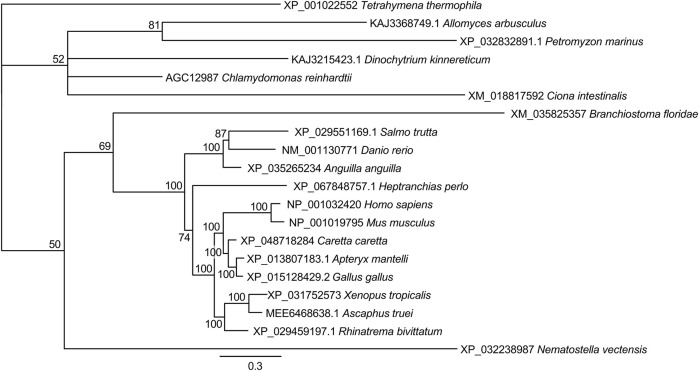
Phylogeny of the C-terminal tail of CDKL5 proteins. The alignment of the C-terminal regions beyond the kinase domain was used to construct a phylogenetic tree by the Bayesian method. The scale bar indicates substitutions per site. For each branch, the bootstrap statistical support is indicated as a percentage. For each sequence, the accession number and species are indicated.

Among the CDKLs, CDKL5 proteins have relatively large C-terminal regions, and in CDD patients, mutations in the kinase domain and in the C-terminus tend to differ (Hector et al., 2017). The C-terminal region seems to be important for protein stability and localization (Lin, Franco, and Rosner, 2005; Rusconi et al., 2008), although it may have further roles. To shed light on the conservation of the C-terminal region and to elucidate which models would be adequate to study it, we also built a phylogeny of these regions of all the CDKL5 proteins identified in our previous kinase domain phylogeny. In addition, we included the C-terminal region of the zoosporidic CDKL protein of uncertain origin. Since it was the best method in the first phylogeny, we decided to use the Bayesian method, but in this case, we were not able to identify a sequence that could be used as an outgroup. Therefore, the resulting tree was unrooted (Figure 3). In this tree, the only clades that had a consistently robust bootstrap were those of Gnathostomata, that is, all the vertebrates with the exception of jawless fish. The remaining organisms could not be resolved into branches or had low bootstrap values. Moreover, the pattern of the vertebrate sequences faithfully reproduced the accepted phylogeny for these species, which was indicative of conservation. Close inspection of the alignments revealed several regions with clear conservation with interspersed variable regions. Therefore, the C-terminal tail of CDKL5 only seems to have selective pressure in jawed vertebrates.

### 3.4 The *Drosophila Cdkl* gene

**FIGURE 4:**
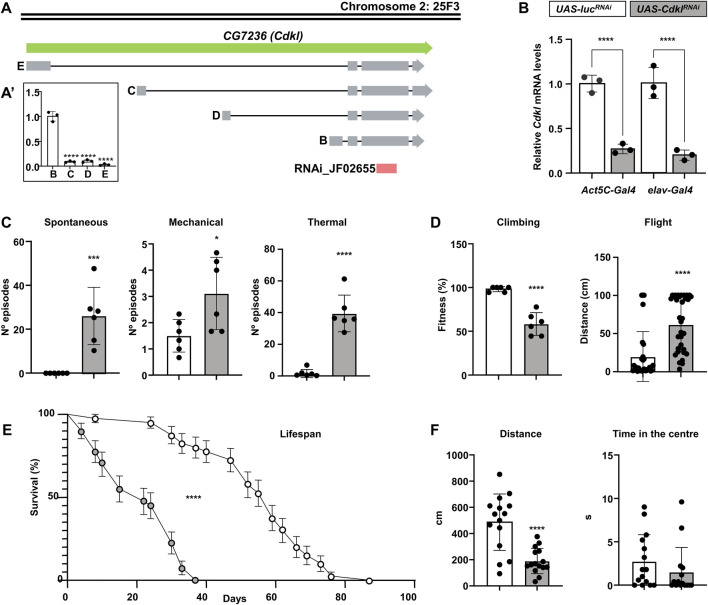
Structure and function of the *CG7236* (*Cdkl*) locus. **(A, A′)** Annotated transcriptional isoforms of the *CG7236* gene; exons are indicated by grey boxes, introns are indicated by solid lines, and the region targeted by JF02655 RNAi is indicated by a red box **(A)**. Relative expression level of the four isoforms **(A′)**. **(B)** Reduction of *Cdkl* transcript levels by driving the expression of *UAS-Cdkl* ^*RNAi*^ (grey) via ubiquitous *Act5C-Gal4* or pan-neuronal *elav-Gal4*, compared with an irrelevant *UAS-luc* ^*RNAi*^ (white); color codes are maintained throughout the figure. **(C)** Increased susceptibility to spontaneous and mechanically and thermally induced seizures (number of episodes) in *elav>Cdkl* ^*RNAi*^ individuals. **(D)** Decrease in negative geotaxis (% flies reaching the 8 cm mark) and flight stabilization (distance until stabilization) in *elav>Cdkl* ^*RNAi*^ individuals. **(E)** *elav>Cdkl* ^*RNAi*^ flies have a shortened lifespan. **(F)** Behavioural alterations in *elav>Cdkl* ^*RNAi*^ individuals in a closed arena regarding total distance walked and the proportion of time spent in the centre. Statistics: **(A′, B)** ANOVA, Šídák’s multiple comparisons test; **(C,D,F)** Student’s t-test, except Flight and Time in the Centre, Mann‒Whitney; **(E)** Log-rank (Mantel‒Cox) test; bars represent mean ± SD (**p < 0.01; ***p < 0.005; ****p < 0.0001).

There is a single gene in *Drosophila* that is homologous to the human *CDKL* gene, annotated as *CG7236*, which is most similar to *CDKL1*. In light of the evidence from our results, which validated its functional equivalence with the *CDKL* family, more specifically with *CDKL5* in human neurons, we propose to name it *Cdkl* following standard procedures in *Drosophila* genetic nomenclature. Even if no specific studies have been conducted on *Cdkl*, we used FlyBase (release FB2024_04) to find information on gene structure and expression (Jenkins et al., 2022). *Cdkl* is most strongly expressed in the head, thoracic-abdominal ganglion (part of the central nervous system) and eye (peripheral nervous system). Automatic annotation predicts four alternative transcripts due to different origins of transcription, as they differ only in the first exon (Figure 4A). The kinase domain starts in the second exon, which is common to all of them. High-throughput studies available through FlyBase suggest that expression levels are low compared with those of other genes and that the predominant isoform is B, but there are no cDNAs or ESTs corresponding to this gene, probably owing to the low level of expression. To test this hypothesis, we performed “mock” quantitative PCR with forward primers specific for the first exon of each isoform and a common reverse primer, using head RNA extracts (Figure 4A’). This is not a rigorous experiment in expression levels, since only one of the primers is common to all experiments, but it could give us an idea of the relevant transcript. The results confirmed that isoform B is the most abundant isoform in the head extracts, which contained mostly neural tissue. Subsequent rescue experiments with this isoform would also support the notion that this is the relevant isoform (see below).

We obtained a stock from the Transgenic RNAi Project (TRiP) (Zirin et al., 2020) bearing an RNAi targeting the *Cdkl* gene under the Gal4-inducible UAS promoter (*UAS-Cdkl* ^*RNAi*^, Figure 4A). For the subsequent experiments with this line, we used an irrelevant RNAi against luciferase (*UAS-luc* ^*RNAi*^) inserted in the same *attP* site as a negative control. We drove the expression of *UAS-Cdkl* ^*RNAi*^ under ubiquitous *Act5C-Gal4* and panneuronal *elav-Gal4* and extracted RNA from the fly heads to perform qPCR (Figure 4B). In both cases, we detected a similar reduction in the number of *Cdkl* transcripts to approximately 20%–25% of the normal level, which is consistent with the predominant neuronal expression of this gene. Therefore, all the phenotypic characterization was performed with the *elav-Gal4* driver, including a *UAS-Dcr2* construct to increase efficiency (*elav>Cdkl* ^*RNAi*^).

In *Drosophila* models of genetic epilepsies, the standard strategies to induce seizures are mechanical shock by vortexing and thermal shock by immersion in a hot water bath (Fischer et al., 2023; Mituzaite et al., 2021). In addition to these, we also determined whether *Cdkl* downregulation caused spontaneous seizures. *elav>Cdkl* ^*RNAi*^ flies presented convulsive phenotypes (Figure 4C). In the absence of any stimulus, they experienced short spontaneous seizures scored over a period of 3 min, similar to the myoclonic seizures and spasms predominant in CDD patients, and they displayed compulsive and repetitive grooming behavior reminiscent of the patients’ stereotypies (Supplementary Video S1). Compared to control flies, seizures were moderately increased by mechanical stress, but this increase was dramatic by heat shock. Strikingly, even after the heat shock, flies continued to have seizures and repetitive grooming behavior for some time (Supplementary Video S2).

With respect to motility, both climbing and flying proficiency were greatly lower in *elav>Cdkl* ^*RNAi*^ flies than in control flies (Figure 4D). Lifespan was also reduced in terms of both life expectancy and maximum lifespan (Figure 4E). When individual flies were followed in a closed arena, they moved less than half than control flies did; however, they did not spend more time in the center of the arena (Figure 4F).

### 3.5 *Drosophila Cdkl* and human *CDKL5* can rescue the *elav>Cdkl* ^*RNAi*^ phenotypes

For the rescue experiments, we generated UAS constructs for the expression of fly *Cdkl* and human *CDKL5*. For *UAS-Cdkl*, since there are no cDNAs available, we amplified a genomic fragment including the coding and intronic regions of isoform B into the *pUASattB* plasmid and generated independent transgenic lines by insertion in the attP sites ZH-51D and ZH-86Fb in chromosomes two and 3, respectively. For *UAS-CDKL5,* we used a human cDNA clone following the same procedure.

**FIGURE 5:**
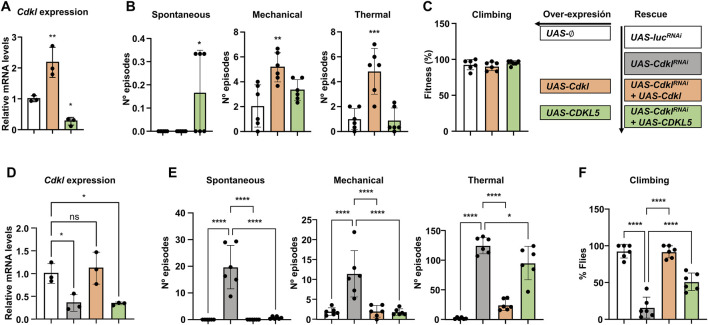
Rescue with *Cdkl* and *CDKL5*. **(A)** Effects of *elav-Gal4*-directed expression of *UAS-Cdkl* and *UAS-CDKL5* on the transcript levels of *Cdkl* in head extracts compared with the empty *attP* insertion site. **(B)** Susceptibility to spontaneous, mechanically and thermally induced seizures upon expression of *UAS-Cdkl* and *UAS-CDKL5*. **(C)** Negative geotaxis in the same genotypes. **(D)** Comparison of *Cdkl* transcript levels in control flies (*UAS-luc* ^*RNAi*^), *UAS-Cdkl* ^*RNAi*^, and the rescues with *UAS-Cdkl* and *UAS-CDKL5*. **(E)** Rescue of the seizure phenotypes of *elav>Cdkl* ^*RNAi*^ by coexpressing *Cdkl* and *CDKL5*. **(F)** Rescue of the negative geotaxis phenotypes of *elav>Cdkl* ^*RNAi*^ by coexpressing *Cdkl* and *CDKL5*. Statistics: ANOVA in all panels; multiple comparisons tests **(A–C)** Dunnett’s test and **(D–F)** Šídák’s test; bars represent mean ± SD (*p < 0.05; **p < 0.01; ***p < 0.005; ****p < 0.0001).

First, we ensured that the transgenic constructs could be expressed under an *elav-Gal4* driver, and we performed these experiments with insertions in the *attP ZH-51D* site. With respect to the *Cdkl* expression levels, we performed quantitative PCR experiments using the empty *attP ZH-51D* as our baseline expression reference (*UAS-∅*) and *elav>Cdkl* and *elav>CDKL5* as our experimental genotypes (Figure 5A). As expected, *elav>Cdkl* flies had elevated transcript levels, approximately twofold greater than those of the control, but *elav>CDKL5* flies presented reduced *Cdkl* transcript levels. This last result suggested that *Cdkl* expression must be subject to a negative feedback loop, so excessive function decreases gene expression. *CDKL5* expression was corroborated only in *elav>CDKL5* flies by RT‒PCR and agarose gel electrophoresis (not shown); in this case, quantitative PCR was not useful since there was no baseline reference. Next, we determined that the expression of these constructs alone had no detrimental effects on seizures or motor phenotypes (Figure. 5B,C). The expression of *CDKL5* slightly increased in spontaneous seizures, and the expression of *Cdkl* had the same effect on heat- and mechanically induced seizures. In any case, these effects were almost irrelevant compared with the number of episodes caused by *elav-RNAi* (Figure 4C). None of them had any effect on negative geotaxis. Therefore, we could conclude that these constructs did not have any serious detrimental effects.

Once we ensured that both constructs were expressed and had no major phenotypic impact, we proceeded with the rescue experiments, using four genotypes, all with *elav-Gal4* but with different UAS constructs. To eliminate genetic background effects, our wild-type control was the same *UAS-luc* ^*RNAi*^ used in previous experiments (Figure 4). The two rescue genotypes contained *UAS-Cdkl* ^*RNAi*^ together with either *UAS-Cdkl* or *UAS-CDKL5*. First, we also checked the *Cdkl* expression levels in all four genotypes (Figure 5D). The expression of *Cdkl* ^*RNAi*^ decreased the transcript level by approximately 65%, and the coexpression of *Cdkl* restored it to the wild-type. In contrast, coexpression of *CDKL5* did not have any effect; therefore, any phenotypic rescue we may observe in this genotype would not be due to *Cdkl* expression. With respect to seizure phenotypes (Figure 5E), both *Cdkl* and *CDKL5* were able to fully suppress spontaneous and mechanically induced seizures to wild-type levels; in heat-induced seizures, Cdkl expression also resulted in a full recovery, while CDKL5 was also able to reduce them but less efficiently. Regarding negative geotaxis, both transgenic strains were also able to rescue the phenotype (Figure 5F). In summary, *Cdkl* can rescue all the phenotypes observed in *Cdkl* ^*RNAi*^, validating this strategy; moreover, human *CDKL5* can rescue the same phenotypes with identical or comparable efficiency, which supports its use as a model for CDD with respect to the phenotypes caused by mutations in the kinase domain.

## 4 Discussion

### 4.1 The origin and diversification of CDKL proteins

CDKL proteins can be found in all major eukaryotic groups, which means that they must have been present in the LECA. In the phylogeny of the kinase domain, the CDKL proteins of Ciliophora and green algae group with vertebrate CDKL5, suggesting that the original CDKL protein had a kinase domain that was most similar to that of extant CDKL5 proteins. Our search also revealed that CDKL proteins were absent in those clades that did not possess cilia or flagella, which are also ancestral eukaryotic organelles (Carvalho-Santos et al., 2011). Among Viridiplantae, they were present in Chlorophyta but absent in Magnoliophyta; among fungi, they were present in Zoosporidic species but absent in the rest, and they were also missing in the Filasterea ameboid *C. owczarzaki*. This finding implies that the ancestral CDKL protein was involved in ciliary/flagellar functions. In fact, this role is maintained in extant CDKL proteins: *C. reinhardtii* mutant for CDKL5 display elongated flagella (Tam et al., 2013); *C. elegans* CDKL-1 localizes to the ciliary transition zone, and mutations in this gene also produce elongated cilia (Canning et al., 2018); and both cultured CDKL5 knockdown cultured rat neurons and CDKL5 mouse knockout *in vivo* neurons also have elongated primary cilia (Di Nardo et al., 2022). Thus, the original role of CDKL proteins was restricting cilium/flagellum length. This does not mean that this is the only function of this protein family; it is evident that during evolution, they may have acquired further roles through the phosphorylation of an extended range of targets. In the case of CDKL5, phosphorylation targets include microtubule-binding proteins (Baltussen et al., 2018; Munoz et al., 2018), transcriptional regulators (Khanam et al., 2021; Kim et al., 2020), and the voltage-gated calcium channel Cav2.3 (Sampedro-Castaneda et al., 2023).

Clearly, CDKL proteins have acquired further targets beyond the cilium; still, the question remains whether this ancestral ciliary function is somehow related to CDD pathology. Cilia in the ependymal epithelia of *Cdkl5* knockout mice are elongated and move asynchronously, resulting in an altered flow of cerebrospinal fluid in the third ventricle of the brain (Faubel et al., 2022). Therefore, we cannot discard that ciliary motility also contributes to the pathophysiology of CDD, although it may not be the main cause.

The single original CDKL protein-encoding gene underwent successive duplications. Although this protein was most similar to CDKL5, the single CDKL proteins of zoosporidic fungi form a separate branch in the phylogeny, which is closer to the CDKL1/2 branch. Two alternative scenarios are that a first duplication occurred in the fungal ancestor, followed by loss of CDKL5, or that the ancestral CDKL5 evolved at a fast pace to adapt to particularities of fungal biology. Since the available evidence is not enough to support either of these two hypotheses, we have grouped these sequences as CDKL-F.

Furthermore, there were proteins similar to CDKL1 in the unicellular Choanoflagellata and in the primitive placozoan animals, although the latter had lost CDKL5 and proteins similar to CDKL1 and CDKL2 in cnidarians and most bilaterians, so the order of appearance must have been CDKL1 and then CDKL2. These two branches group together and separately from CDKL5, so CDKL2 most likely arose as a duplication from CDKL1. A dramatic reduction in the expression of both CDKL2 and CDKL5, which leave CDKL1 to perform all the required functions, is observed in Ecdysozoa. A second, rapidly diverging CDKL protein appeared in insects (CDKL-I), although it was eventually lost, at least in Diptera. Therefore, except for Ecdysozoa, most animals contain a basic set of CDKL1/2/5 proteins, and the ancestor of Vertebrata has undergone at least two rounds of duplication of CDKL1.

The next round of duplication and differentiation occurred in the ancestor of Gnathostomata, with CDKL1 giving rise to CDKL4 and CDKL2 giving rise to CDKL3. These animals would already have representatives of all five classes of CDKL proteins, with duplications of CDKL1, which were subsequently lost. The final set of five CDKL proteins that we observe in humans was already present in the ancestor of bony fish and vertebrates, although during evolution, one or more members were lost in the different classes or even at lower levels within a class.

### 4.2 CDKL proteins can be functionally equivalent

During evolution, CDKL-encoding genes have undergone several events of gene duplication. The new copies were probably used to provide the function in different tissues, developmental stages, or physiological conditions. But it is also true that there are numerous events of gene loss, in addition to those cases associated with the loss of cilia and flagella: loss of CDKL5 in Placozoa, loss of CDKL2 and CDKL5 in Ecdysozoa, CDKL-I in Diptera, or a number between one and three in all or part of certain vertebrate classes. This means that either those genes were redundant or that the remaining genes could provide for the lost function. In CDKL proteins, the kinase domain is highly conserved, and there are no additional domains; thus, these domains are likely equivalent at the molecular and functional levels. In agreement with these findings, the overexpression of endogenous CDKL-1 and human CDKL5 in *C. elegans* restricts cilium length, and pathological mutations in CDD patients introduced in CDKL-1 abolish this ability (Canning et al., 2018). In our work, we also demonstrate that the deficiency of *D. melanogaster* Cdkl, which is also similar to CDKL1, can be corrected by the expression of human CDKL5.*Drosophila Cdkl* knockout flies display mutant phenotypes very similar to those we describe in this work, and, remarkably, these phenotypes are rescued by expression of human CDKL5, CDKL1 and CDKL2, representing the three main branches of the CDKL protein family (Bereshneh et al., 2025). The aforementioned observations strongly support the notion of functional redundancy of the kinase domain of CDKL proteins.

Therefore, the main difference among the different CDKL-encoding genes would be the transcriptional regulation of their expression, rather than the function they encode. This would explain why the full phenotypical manifestation of CDKL5 mutations in model organisms could be masked by leaky expression of other family members. In fact, it has been demonstrated that mouse CDKL2 can phosphorylate CDKL5 targets, reinforcing the notion of functional redundancy (Silvestre et al., 2024).

In contrast to the kinase domain, the C-terminal region of CDKL5 is conserved only in jawed vertebrates. This region is considerable in length, with up to several hundred amino acids. The phylogenetic tree shows that these regions are subject to selective pressure, and the alignment reveals that there are short and interspersed stretches with a relatively high level of conservation. This region is poorly understood, but it is clearly important for controlling protein levels, autophosphorylation activity and sub-cellular localisation (Lin et al., 2005; Rusconi et al., 2008). Consequently, the clinical manifestation of mutations in this region is different from that of mutations in the kinase domain, and they tend to produce milder symptoms (Diebold et al., 2014; Hector et al., 2017). Since the C-terminal region is conserved only in vertebrates, mutations in it can only be studied in vertebrate biomedical models. Interestingly, the acquisition of a distinct function for this C-terminal region coincides with the origin of myelination in placoderms, the first hinge-jawed fish (Zalc et al., 2008), suggesting that it could have a role in this process. In agreement with these findings, some CDD patients display defects in myelination (Bahi-Buisson and Bienvenu, 2012), and mouse models of CDKL5 also exhibit hypomyelination in the cortex (Maurizio Giustetto, personal communication).

### 4.3 Invertebrates can be a model for CDD caused by mutations in the kinase domain

In this work, we generated a CDD model in *Drosophila* by RNAi-mediated downregulation of *Cdkl* in all neurons. *Ckdl* ^*RNAi*^ flies had a phenotype that is reminiscent of many of the clinical features of CDD patients. They suffered spontaneous seizures of the myoclonic type, brief and shock-like, and compulsive grooming, which is different from the normal organised, sequential grooming (Seeds et al., 2014) and akin to patients’ stereotypies. The frequencies of seizures and compulsive grooming are greatly increased after heat shock and are more modest upon mechanical stress. In addition, these flies also had lower competence in climbing and flight stabilization and less mobility in an open arena.

These phenotypes seem to be rather specific, especially when we compare them with other seizure models. A *Drosophila* Dravet syndrome model developed in our laboratory also displayed predominantly heat-induced seizures, but these were of the tonic‒clonic type; they did not appear in the absence of the stimulus, and the individuals had no stereotypical behaviour. Although this model also had reduced motility and motor competence, it also had a tendency to spend more time in the centre of the open arena, suggesting a cognitive impairment (Tapia et al., 2021), which did not occur in our CDD flies.

All these phenotypes were suppressed by co-expression of *Cdkl*, demonstrating that they are due to the downregulation of this gene and not to off-target or non-specific effects. Most importantly, they were almost completely suppressed also by co-expression of human *CDKL5* cDNA, demonstrating that the insect Cdkl and human CDKL5 proteins can perform the same molecular functions. The slightly lower efficiency of CDKL5 compared to Cdkl could be due to the presence of the large C-terminal tail, which is not present in the fly protein. Before we performed these rescue experiments, we ensured that the expression of *UAS-Cdkl* and *UAS-CDKL5* did not have any detrimental effects by themselves. We detected only very mild increases in seizure susceptibility, but interestingly, we observed that endogenous *Cdkl* expression was reduced upon expression of CDKL5. This effect suggests that Cdkl expression is subject to a negative feedback loop at the transcript level to prevent excessive function, which could be detrimental to the individual.

Human CDKL5 was also expressed in *C. elegans*, and it shared the ability to restrict cilium length with endogenous CDKL-1 (Canning et al., 2018). The difference from the current work is that those results were based on the overexpression (gain of function) of the proteins, whereas in our case, we focused on the suppression of the loss of function by the expression of the human protein. All this evidence strongly suggests that invertebrate models can be informative in the research of CDKL5 functions residing in its kinase domain and the effects of mutations in CDD patients.

## 5 Conclusion

CDKLs are ancestral eukaryotic proteins that play a role in the maintenance and/or function of cilia and flagella, although their phosphorylation targets may have diversified during evolution to regulate other cellular functions. The kinase domain is conserved across all eukaryotic species, and the pattern of gene gain and loss suggests that the different family members are functionally equivalent at the molecular level, probably due to the conservation of the kinase domain. The fact that we could rescue the downregulation of *Drosophila* Cdkl with expression of human CDKL5 supports this hypothesis. The *Drosophila* model we have generated will be a valuable tool in the research towards understanding and treating CDD, based on the phenotypes and the aforementioned rescue. On the other hand, the C-terminal tail has defined functions only in jawed vertebrates; therefore, only vertebrate animal and cellular models would be relevant to study this region.

## Data Availability

The protein sequence alignments have been deposited in Zenodo (https://doi.org/10.5281/zenodo.13904044). The raw data of all experiments and the precise statistical significance can be found in the article/Supplementary Material.
