# Supplementary material for: A phylogenetic analysis of the CDKL protein family unravels its evolutionary history and supports the Drosophila model of CDKL5 deficiency disorder
Source: Front Cell Dev Biol. 2025 Apr 30;13:1582684. doi: 10.3389/fcell.2025.1582684 (PMC12075339; doi:10.3389/fcell.2025.1582684)
Supplement: Supplementary file 1 [file DataSheet1.zip › Figure S1.PDF]

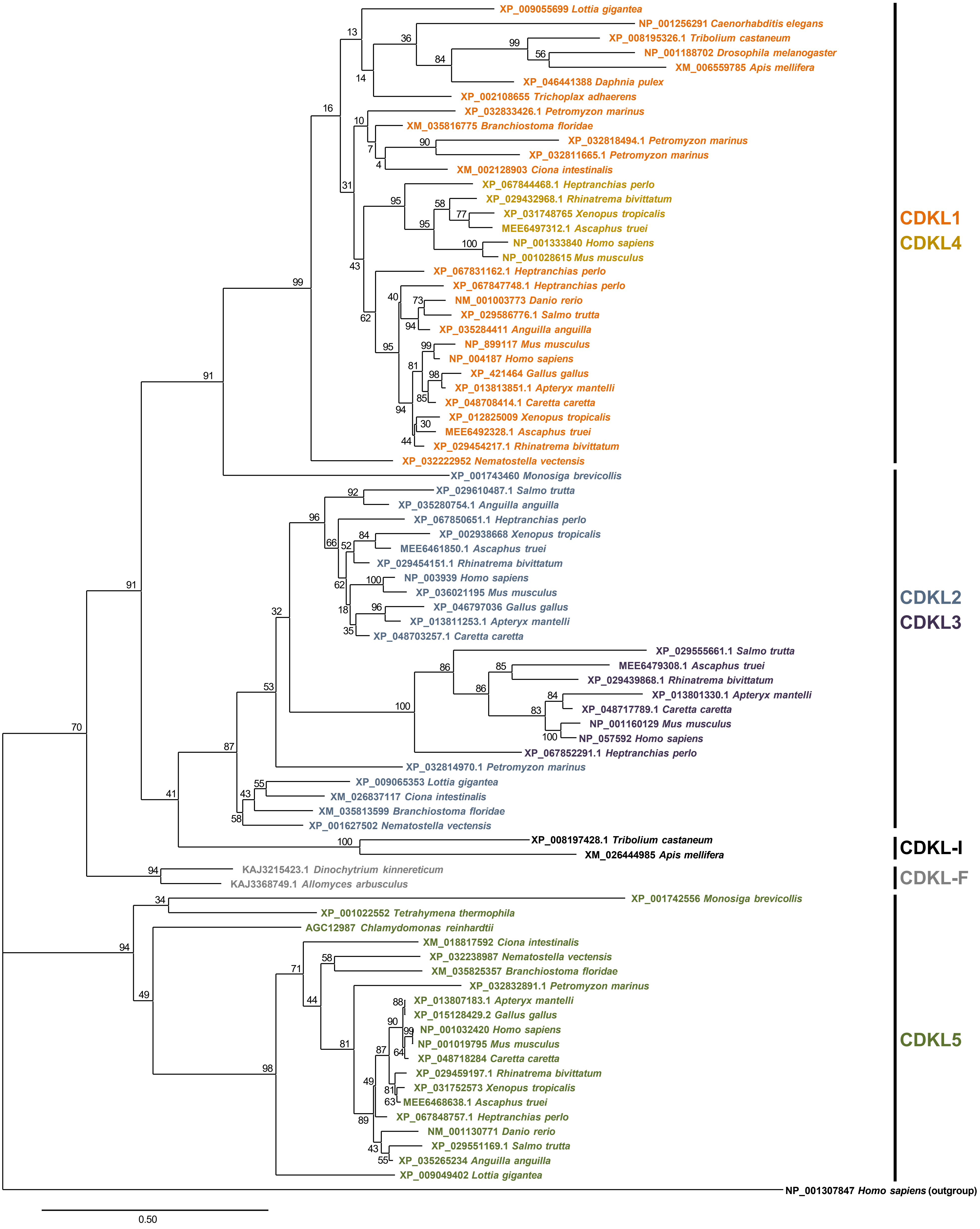

Figure S1. Maximum likelihood phylogenetic tree of the kinase domain of CDKL proteins. Branch lengths are proportional to the amount of genetic change measured as substitutions per site, the scale bar is shown below the tree. For each branch the bootstrap statistical support is indicated as percentage. For each sequence, the accession number and species are indicated. On the right we indicate the protein families defined on the basis of the phylogeny.
