# Supplementary material for: A phylogenetic analysis of the CDKL protein family unravels its evolutionary history and supports the Drosophila model of CDKL5 deficiency disorder
Source: Front Cell Dev Biol. 2025 Apr 30;13:1582684. doi: 10.3389/fcell.2025.1582684 (PMC12075339; doi:10.3389/fcell.2025.1582684)
Supplement: Supplementary file 1 [file DataSheet1.zip › Tables S1-2.pdf]

Table S1. *Drosophila* stocks employed in this study. B# refers to the Bloomington *Drosophila* Stock Center stock number.

ORIGIN	GENOTYPE	NAME
B#31603	y^{1} v^{1}; P{$y^{+t7.7}$ $v^{+t1.8}$=TRiP.JF01355}attP2	*UAS-luc^{RNAi}*
B#27505	y^{1} v^{1}; P{$y^{+t7.7}$ $v^{+t1.8}$=TRiP.JF02655}attP2	*UAS-Cdkl^{RNAi}*
B#458	P{$w^{+mW.hs}$=GawB}$elav^{C155}$	*elav-Gal4*
B#25750	P{$w^{+mW.hs}$=GawB}$elav^{C155}$; P{w^{+mC}=UAS-Dcr-2.D}2	*elav-Gal4, UAS-Dcr-2*
B#3954	y^{1} w*; P{w^{+mC}=Act5C-GAL4}17bFO1/TM6B, Tb^{1}	*Act5C-Gal4*
B#24483	y^{1} M{$RFP^{3xP3.PB}$ $GFP^{E.3xP3}$=vas-int.Dm}ZH-2A w*; M{3xP3-RFP.attP}ZH-51D	*attP-ZH-51D*
B#24749	y^{1} M{$RFP^{3xP3.PB}$ $GFP^{E.3xP3}$=vas-int.Dm}ZH-2A w*; M{3xP3-RFP.attP}ZH-86Fb	*attP-ZH-86Fb*
This work	w*; M{w^{+mC}=UAS-hCDKL5}ZH-51D	*UAS-CDKL5*
This work	w*; M{w^{+mC}=UAS-Cdkl}ZH-51D	*UAS-Cdkl*

Table S2. Sequences of all the primers used in the molecular biology procedures.

Adapters for restriction enzyme cloning are indicated in lowercase.

NAME	SEQUENCE	PROCEDURE
pUAST-Cdkl-F	GGGAATTCGTTAACAGATCTGCGGCCGCG ACAGCCCTTGAAATAATACCAGATCG	Gibson cloning *Cdkl*
pUAST-Cdkl-R	AGGTTCCTTCACAAAGATCCTCTAGACAG GACGCAACTGGAAAGTGA	Gibson cloning *Cdkl*
pUAST-CDKL5-F	ggaattgggaattcgttaacaGATCCGGT ACCGAGGAGATCTGCC	Gibson cloning *CDKL5*
pUAST-CDKL5-R	gttccttcacaaagatcctCTCTAGATCT GTTCAGGAAACAGCTATGACCG	Gibson cloning *CDKL5*
LoxP-F	CTTCGTATAATGTATGCTATACGAAG	PCR *UAS-Cdkl* and *UAS-CDKL5* transgenics
Cdkl-Lox-P-R	GGCTTACCTTCAGTAGCCTAATTTC	PCR *UAS-Cdkl* transgenics
CDKL5-Lox-P-R	GCATTATTGCCTTCTGACAGATT	PCR *UAS-CDKL5* transgenics
Rpl32 F	ATGCTAAGCTGTCGCACAAATG	qPCR *Cdkl*
Rpl32 R	GTTCGATCCGTAACCGATGT	qPCR *Cdkl*
FoxK/Mnf Fwd	GAGCAGAAGAGCCCCTACCT	qPCR *Cdkl*
FoxK/Mnf R	AATGAAACCCTGACGTGGAC	qPCR *Cdkl*
eEF1α1 F	GCGTGGGTTTGTGATCAGTT	qPCR *Cdkl*
eEF1α1 R	GATCTTCTCCTTGCCCATCC	qPCR *Cdkl*
Cdkl-B-F	CACATGTGTTCGTGTCTGAGGTATC	qPCR *Cdkl*,<br>RT-PCR *Cdkl* isoforms
Cdkl-C-F	TCACAGCTCATCGTTCTACTTGC	RT-PCR *Cdkl* isoforms
Cdkl-D-F	CGTCTTCACATCGTGTAGTTCTGC	RT-PCR *Cdkl* isoforms
Cdkl-E-F	TGCGGAAATCACTTTCGAGATTCCA	RT-PCR *Cdkl* isoforms
Cdkl-R	CACTTGTAGACCACACCGTAGGAG	qPCR *Cdkl*,<br>RT-PCR *Cdkl* isoforms
CMV-CDKL5-Seq1-F	AGGCAATAATGCTAATTACA	Sequencing *CDKL5*
CMV-CDKL5-Seq1-R	GAGATCTAGAGTTCAGGAAACAGCTATGA	Sequencing *CDKL5* from CMV
CMV-CDKL5-Seq2-F	AGTCTCACCACAGATCTAA	Sequencing *CDKL5*
CMV-CDKL5-Seq2-R	GAATCCGAATTTCTGAGAA	Sequencing *CDKL5*
CMV-CDKL5-Seq3-R	TAGGTGTCTATTTTCTTTGG	Sequencing *CDKL5*

pUAST-Cdkl -A-F	ACCTGACCAAACAGATCTG	Sequencing
pUAST-CDKL5-int-A- F	ATGGACAGCCTTTATTTCCTG	Sequencing pUAST-CDKL5
pUAST-CDKL5-int-B-F	CGGCATAGCTATATTGACACA	Sequencing pUAST-CDKL5
pUAST-CDKL5-int-R	GTCTGCAGATCTGAGATCT	Sequencing pUAST-CDKL5
pUAST-CDKL5-Seq-F	GTAACCAGCAACCAAGTAAATC	Sequencing pUAST-CDKL5
pUAST-CDKL5-Seq-R	GCTTTAAATCTCTGTAGGTAG	Sequencing pUAST-CDKL5
pUASTattB-F	GTAACCAGCAACCAAGTAAATC	Sequencing pUASTattB clones
pUASTattB-R	GCTTTAAATCTCTGTAGGTAG	Sequencing pUASTattB clones
pUAST-Cdkl-2R/3R-R	TGGTCTGCAAGGAAGTTAACA	Sequencing *UAS-Cdkl* transgenics
pUAST-CDKL5-2R	AATGCTTCCTTCAACTCCACAATG	Sequencing *UAS.CDKL5* transgenics
